# Prevalence and Determinants of Rural-Urban Utilization of Skilled Delivery Services in Northern Ghana

**DOI:** 10.1155/2020/9373476

**Published:** 2020-05-10

**Authors:** Mahama Saaka, Jones Akuamoah-Boateng

**Affiliations:** School of Allied Health Sciences, University for Development Studies, P.O. Box TL 1883, Tamale, Ghana

## Abstract

**Background:**

There are wide differences in the uptake of skilled delivery services between urban and rural women in the northern region of Ghana. This study assessed the rural-urban differences in the prevalence of and factors associated with uptake of skilled delivery in the northern region of Ghana.

**Methods:**

The study population comprised postpartum women who had delivered within the last three months prior to the study. The dataset was analyzed using the chi-square test and multivariable logistic regression.

**Results:**

The odds of skilled birth attendance (SBA) adjusted for confounding variables in urban areas were higher compared with their rural counterparts (AOR = 1.59; CI: 1. 07–2.37; *p*=0.02). The determinants of skilled delivery were similar but of different levels and strength in rural and urban areas. The main drivers that explained the relatively high skilled delivery coverage in the urban areas were higher frequency of antenatal care (ANC) attendance, proximity (physical access) to health facility, and greater proportion of women attaining higher educational level of at least secondary school. Distance from health facility less than 4 km was the greatest independent contributor to the variance in skilled delivery in the urban areas, whereas frequency of ANC attendance was the greatest independent contributor in the rural areas.

**Conclusions:**

This study identified underlying determinants accounting for rural-urban differences in skilled delivery, and covariate effect was more dominant than coefficient effect. Therefore, urban-rural differences in SBA outcomes were primarily due to differences in the levels of critical determinants rather than the nature of the determinants themselves. Therefore, improving skilled delivery outcomes in this study population and other similar settings will not require different policy frameworks and interventions in dealing with rural-urban disparities in SBA outcomes. However, context-specific tailored approaches and strategies including targeting mechanisms have to be designed differently to reduce the rural-urban differences.

## 1. Introduction

Maternal mortality ratio (MMR) in Ghana is still a big problem as the WHO puts the current MMR estimates at 319 maternal deaths per 100,000 live births [1]. The current situation shows that a lot more have to be made in order to achieve the Sustainable Development Goal-3 (SDG-3) [2].

Though facility-based deliveries in Ghana have increased from 42% in 1988 to 73% in 2014, skilled delivery care service utilization is still below set targets. The 2014 Ghana Demographic and Health Survey (GDHS) indicated that nearly three-quarters of births (74.0%) in Ghana occurred with the assistance of a skilled health professional.

Efforts to make skilled birth services available to pregnant women in Ghana started in 2005 when the Government of Ghana implemented a number of social interventions including the nationwide free maternal health services policy, the National Health Insurance Scheme (NHIS), and the Maternal Healthcare Program [3].

In spite of all these strategic measures, maternal mortality still remains a risk factor for women in Ghana, especially in rural areas where utilization of skilled delivery services is reported to be 59.0% among rural women compared to 90.0% in urban areas [4, 5].

One potential intervention that could help reduce maternal mortality and improve perinatal outcomes for newborns is pregnant women seeking skilled assistance during childbirth.

A skilled birth attendant (SBA) as defined by the WHO is someone who has been “trained to proficiency in the skills needed to manage normal (uncomplicated) pregnancies, childbirth and the immediate postnatal period, and in the identification, management and referral of complications in women and newborns” [6].

It is estimated that, if there were skilled birth attendants (SBAs) at all deliveries, maternal mortality could be reduced by 13–33% [7]. SBA rate therefore serves as an indicator of progress towards reducing maternal mortality worldwide [8, 9].

Though skilled assistance during childbirth is a critical strategy for reducing maternal mortality [6, 10, 11], the proportion of deliveries taking place within health facilities is below expectations especially in Northern Ghana, with huge disparity between urban and rural women. For example, in the year 2012, the annual report of Ghana Health Service showed that uptake of skilled delivery services in the Tamale Metropolis (urban area) and the Nanumba North District (rural setting) was 67% and 36%, respectively.

The difference in skilled assisted deliveries raises the question as to whether different policies and interventions are required in these areas. Differences may arise because of differences in the levels of determinants of skilled delivery outcomes (covariate effects) or differences in the strength of association between particular determinants and delivery outcomes (coefficient effects), or differences may also arise from a combination of covariate and coefficient effects. If the differences arise largely due to covariate effects, similar policy frameworks and tools could be applied [12], but if differences are largely due to coefficient effects, then different strategies may be needed.

Information on the determinants of the rural-urban differentials in uptake of skilled delivery services is relevant since better policies, programs, and strategies that are necessary to improve skilled deliveries may differ in rural and urban areas. Eventhough there are wide disparities in the use of skilled delivery services between urban and rural women in the Northern Region of Ghana, information on factors responsible for these disparities has not been adequately explained. Furthermore, understanding the nature and relative importance of the various determinants of skilled delivery in different settings is key to designing effective context-relevant program and policies tailored to the needs of each setting. Against this background, this study assessed the structural differences in the determinants of uptake of skilled birth delivery services and their strength of association across urban and rural districts of Northern Region of Ghana.

## 2. Materials and Methods

### 2.1. Study Location

The study was conducted in Nanumba North District which is largely rural and Tamale Metropolis which is essentially urban. “Urban” and “rural” settlements classification was based on population size. Localities with 5,000 or more persons were classified as urban while localities with less than 5,000 persons were classified as rural [13].

The Nanumba North District was created as a separate district in 2004 under LI 1754 of Ghana from the then Nanumba District which was split into two areas: North and South. The district covers an area of 1,986 sq. km and it is located in the eastern part of the Northern Region and lies between latitudes 8.5°N and 9.25°N and longitudes 0.57°E and 0.5°E.

The Nanumba North District, which is sparsely populated with an annual growth rate of 2.7%, has population of 141,584 (Population and Housing Census, 2010). This population is served by only six health facilities, one of which is a hospital located in district capital at Bimbilla.

The total fertility rate for the district is 3.4. The general fertility rate is 97.5 births per 1,000 women aged 15–49 years. The crude birth rate (CBR) is 22.2 per 1,000 population. The crude death rate for the district is 4.6 per 1,000 [13]. A major health challenge is inadequate health personnel.

The district is predominantly agricultural with about 79.4% of the people engaged in the agriculture, forestry, and fishery sector, followed by those in craft and related trade (6.2%). This basically makes the district economy agrarian [13].

Access to potable water, education, health, electricity, and adequate sanitary facilities is limited and nonexistent in some homes and communities [14]. The quality of life of the people in the district is therefore largely constrained by these facilities.

The Tamale Metropolis was also established under Legislative Instrument (LI) 1801 of 2004.

The population of Tamale Metropolis, according to the 2010 Population and Housing Census, is 233,252 representing 9.4 percent of the region's population.

The metropolis has a total estimated land size of 750 km sq. which is about 13% of the total land area of the Northern Region. Geographically, the metropolis lies between latitudes 9°161 and 9°341 North and longitudes 0°361 and 0°571 West.

The metropolis has a teaching hospital and other hospitals that provide healthcare services to the populace. The total fertility rate for the metropolis (2.8) is slightly lower, compared to the regional fertility rate of 3.5. The general fertility rate is 79.9 births per 1000 women aged 15–49 years. The crude birth rate (CBR) is 21.2 per 1000 population. The crude death rate for the metropolis is 5.6 deaths per 1000 [15].

The occupation with the highest population in the metropolis is service and sales workers (33.0%).

### 2.2. Study Design, Population, and Sampling

A comparative cross-sectional study design was used conducted in the Tamale Metropolis and the Nanumba North District which are located in the northern region of Ghana. The study focused on assessing the uptake of skilled delivery services among rural and urban women.

A sample size of 720 (360 per study area) was used in order to have 80% power of detecting a significant difference of 15% in the primary outcome measure between the urban and the rural groups at 95% confidence interval, assuming a correction factor of 2 (the “design effect”) for cluster sampling. A provision of 10% of total sample size (66) was also factored into the sample size estimation to take care of incomplete/damaged questionnaires.

Postpartum women who had delivered within the last three months prior to the study constituted the study population. A two-stage cluster sampling was used to extract the study population from two districts (one rural and one urban). The study was conducted in 30 clusters each from the Tamale Metropolis and the Nanumba North District. The clusters were selected using probability proportional to size (PPS). All the households in each cluster were serially numbered; the total number of households in a cluster was divided by the sample size to give the sampling interval. The first household was randomly selected by picking any number within the sample interval. Subsequent selections were made by adding the sampling interval to the selected number. This was done until the sample size was obtained.

### 2.3. Data Collection

Structured pretested questionnaires were used to collect quantitative data which included the sociodemographic characteristics of the respondents, maternal history of antenatal care (ANC) utilization, knowledge of complications in labour, uptake of skilled delivery services, perceived quality of care, household decision-making, and household wealth index.

### 2.4. Measurement of Independent and Dependent Variables

The main outcome measure (dependent variable) was utilization of skilled attendance at birth. The independent variables included geographic access (distance to nearest health facility), maternal autonomy in taking decisions that affect mother's health, utilization of antenatal care services and quality of antenatal care services, and sociodemographic characteristics including age of mother, parity, marital status, religion, educational background of mothers, and household wealth index [16].

A brief description of main independent and dependent variables is as follows.

### 2.5. Determination of Proportion of Deliveries Assisted by Skilled Birth Attendants (SBAs)

Skilled attendance rate, the main dependent variable, was measured by asking respondents where delivery of youngest child took place and who assisted with the delivery. A score of 1 was given for delivery by SDBAs (that is, doctor, nurse, or midwife, auxiliary nurse or midwife) while zero (0) was assigned for delivery assisted by persons other than an SBA.

### 2.6. Assessment of Socioeconomic Status

Household wealth index which is a proxy measure of socioeconomic status was used to categorize study participating households. Principal components analysis (PCA) was used to quantify this as per respondent ownership of specified durable goods (television, radio, car, mobile telephone, etc.) and housing characteristics (access to electricity, source of drinking water, type of toilet facilities, type of flooring material, and type of cooking fuel) [17].

### 2.7. Perceived Barriers for Skilled Delivery

To ensure proper utilization of health services, it is critical that potential users of these services have a perceived need for them [18]. It is expected that an increase in the need may lead to an increase in the use of health services and vice versa [18]. Perceived need (self-reported need) of women is based on their belief, previous experience, and the need for skilled health services at birth [18, 19].

Need for skilled health services at birth would be influenced by certain constraints or barriers that the woman perceived. In this study, perceived need for supervised delivery was quantified indirectly by a composite index constructed based on responses to 11 identified key perceived barriers to supervised skilled delivery. A score of 1 was assigned to each barrier if the woman mentioned it as the main reason for not delivering at a health facility. The barriers were no difficulty in previous deliveries, no health facility available, long distance from health facility, bad attitude of health workers, cost of delivery (bed preparedness), lack of privacy during delivery, presence of male staff members during delivery, hospital staffs not allowing women to deliver in their preferred way (squatting), cultural/religious beliefs in conflict with hospital delivery, fear of caesarean delivery, and transportation difficulties.

The total score for each woman was then categorized into low (<median score) or high (≥median score). The assumption is that women with a lower composite barrier score will have a higher need for skilled birth attendance.

### 2.8. Assessment of Content of Antenatal Care (ANC) Services

Women were asked whether specific services including taking of weight and height, measurement of blood pressure, and taking of blood or urine samples were carried out for them. A composite index comprising ten of these essential services received during ANC was created by assigning a score of 1 for having received a particular service and zero for not receiving the service. The index served as a proxy measure of antenatal care quality. The total score for each woman was then categorized as low (<median score) or high (≥median score). The assumption is that women who received high content ANC services will have higher perceived quality of maternal healthcare than those who received lower quality ANC, which is likely to have a positive effect on their use of SBAs.

### 2.9. Assessment of Women's Autonomy

Though a number of dimensions are used in quantifying women's autonomy in the literature [20, 21], three were assessed in this study: decision autonomy, movement autonomy, and maternal financial independence. Each dimension had a number of items that were scored and added to arrive at each respondent's total score.

Women's decision autonomy was estimated from 9 questions on who makes decisions at home; movement autonomy was based on 6 questions on whether women need permission to visit places outside home.

Decision autonomy was estimated from nine questions on decision-making (e.g., children's healthcare, education, buying/selling property, and what to cook) [22]. The responses were scored as follows: 2 points for decisions made by the woman; 1 point for decisions made jointly by both the woman and her husband; and 0 for all of decisions taken by others.

The independent variables for maternal decision-making power include final say on (1) own healthcare, (2) making large household purchases, (3) making household purchases for daily needs, (4) visits to family or relatives, and (5) foods to be cooked each day. The responses were scored as follows: 2 points for decisions made by the woman; 1 point for decisions made jointly by both the woman and her husband; and 0 for all of decisions taken by others.

Movement autonomy was based on 6 questions regarding whether women need permission to visit places outside home (e.g., market, health centre, and relatives' home) [22]. The responses were scored as 1 (no permission required) and 0 (yes, permission always required).

Maternal financial independence was assessed based on four questions that relate to sources of income and savings.

An overall composite index of women's autonomy (CIWA) was calculated by combining the three dimensions. As was done by Singh et al. [23], two categories (that is, low and high) of the individual components and the CIWA were created based on the average value of the index. The women receiving less than the average score were put in the low autonomy category, and those of at least the average score were categorized as high autonomy.

### 2.10. Data Analysis

Data cleaning and analysis were carried out using statistical weighted analysis in SPSS Complex Samples module for Windows 21.0 (SPSS Inc., Chicago). This was done in order to make statistically valid population inferences and computed standard errors from sample data.

The analytical approach used in this study is a modified version of that of Smith et al. [24] which was used to assess rural-urban disparity in child malnutrition. The analysis sought to establish whether the levels of determinants such as mother's educational level (covariate effect) and their strength of association (coefficient effect) with uptake of skilled delivery services differed between rural and urban populations.

The basic assumption in this analysis is that if a determinant, for example, education, is found to be higher in urban than rural areas, but it has a very weak association with the outcome variable (e.g., SBA) in urban relative to rural areas, then it cannot definitively be concluded that education is one of the responsible determinants [24].

In the first step of the analysis, we tested for structural differences in the determinants of skilled birth attendance (SBA) and their strength of association across urban and rural areas. The initial analyses therefore involved estimating the levels of both proximal and distal determinants of SBA in both rural and urban samples. The factors tested are known in the literature to influence uptake of skilled delivery services, and they included socioeconomic status (SES) of mother, antenatal care (ANC) attendance, proximity to health facility, perceived barriers to institutional delivery, and women's decision-making autonomy. For example, what is the proportion of the respondents who had attained higher educational level?

Logistic regression analysis was carried out to estimate the strength of association (coefficient effect) of SBA determinants among urban as well as rural women. Two separate models for urban and rural samples were fitted. The first model (Rural Model) was fitted to identify the determinants of utilization of skilled delivery for rural women. The second model (Urban Model) identified the key determinants of SBA among women living in urban areas.

The same sets of independent variables were used for both the rural and urban areas in order to enable a comparison between both areas. To estimate the strength of association of each determinant with the outcome variable, odds ratio (OR) and 95% confidence interval (CI) were computed.

In the second step of the analysis, we compared the levels of determinants across urban and rural areas, taking into account any structural differences found in the determinants. In comparing the levels of the proximal and distal determinants across rural and urban areas, we tested for significance in the differences in levels across the areas. If the measure of the determinant is continuous, the *t*-test was used for differences in means. If the determinant is categorical, the chi-squared analysis was performed to test for differences in proportions.

The final stage of the analysis involved a comparison of differences in the levels and strength of association of selected determinants of skilled delivery attendance in rural and urban areas. At this stage, a decision was made as to whether the determinant was having a covariate effect, a coefficient effect, or both. To determine whether a factor had “covariate” or “coefficient” effect, we compared both differences in levels as well as strength of association.

If the urban-rural difference of a determinant's level was found to be statistically significant and this determinant was significantly associated with SBA, then that determinant is considered to have both covariate and coefficient effects in the urban setting. On the other hand, if there is significant difference in the level of the determinant but lack of association with the outcome variable, then the determinant will only exhibit covariate effect. If a determinant is associated more significantly with the urban areas than with rural areas but there is no discernible difference in the level of the determinant, then it will be classified as having only coefficient effect.

In the final stage of the analysis, the kind of effect that dominated in the urban areas was assessed by noting how many effects of each kind were shown in the urban area.

### 2.11. Ethics Consideration

The study protocol was approved by the Scientific Review and Ethics Committee of the School of Allied Health Sciences, University for Development Studies, Ghana.

Informed consent was also obtained after providing the required information and explanation. In situations where the respondent could not write or read, verbal informed consent was obtained. In addition, verbal informed consent was sought from all the study participants before the commencement of any interview.

## 3. Results

### 3.1. Sample Characteristics of Respondents

Table 1 shows a comparison of selected background sample characteristics of respondents in the study groups. The study groups were largely comparable except in educational level and religious affiliation where there was significant difference. More Christians were resident in the urban than the rural areas, whereas more people who practice the African traditional religion were concentrated in the rural areas (Table 1).

### 3.2. Skilled Delivery Attendance Coverage

In the whole sample, 45.3% of deliveries were attended by skilled birth attendants (SBAs). Out of the 394 (54.7%) who delivered at home, 67.3% reported delivering all by themselves without the assistance of anybody. Bivariate analysis showed that a greater but insignificant proportion of urban women utilized skilled birth services, compared to rural women (48.6% versus 41.7%), *p*=0.06.

### 3.3. Levels of Utilization of Skilled Delivery Care Services among Women in Rural and Urban Settings (Bivariate Analysis)

Table 2 presents the rural-urban socioeconomic and demographic differentials in the utilization of skilled delivery. The Chi-square association test showed that maternal educational level was not significantly associated with utilization of skilled delivery in urban samples. In the rural areas, only 37.4% of births among women with no education were assisted by SBAs, compared to 75% among those with secondary education or more. In the urban areas, the proportions were 47.1% and 52.5%, suggesting that the percentage difference between women with no education and those with at least secondary education was greater in the rural areas.

Age of the mothers was associated with skilled birth attendance (SBA) in both urban and rural areas. SBA was higher among younger women than their older counterparts. A consistent negative association was observed between parity of the women and use of skilled delivery services across the rural and urban residences.

Use of skilled attendants (SBAs) was high among mothers from households of high wealth index and among mothers whose educational attainment was at least secondary school level (Table 2). Use of SBAs was higher among older women compared to younger ones. Distance to the nearest health facility had a significant influence on SBA. For example, in the rural areas 75.0% of women living within 1–3 km of a health facility gave birth with SBAs compared with 30.2% of women living at least 4 km from a facility. SBA was higher among women with greater autonomy to make their own decisions in both rural and urban areas.

Frequent antenatal care visits (at least 4) increased the use of SBAs at birth, and skilled birth attendance (SBA) rate also increases among mothers who reported 0-1 perceived barrier to institutional delivery compared with those who reported at least 2.

### 3.4. Determinants of Skilled Birth Assistance in Rural and Urban Areas

Table 3 presents results of logistic regression analysis showing determinants of skilled delivery among urban as well as rural women. Two separate models for urban and rural samples were fitted to see the determinants of utilization of skilled delivery services. The first model (Rural Model) was fitted to identify the determinants of utilization of skilled delivery for rural women. The second model (Urban Model) demonstrates the key predictors of utilization of skilled delivery among women living in urban areas.

In the rural areas, the key determinants of SBA were maternal age, high household wealth index, parity, distance from health facility, decision-making autonomy of women, perceived barriers to skilled birth services, frequency of antenatal care (ANC) attendance, and knowledge of danger signs during delivery. In the urban areas, the consistent determinants were parity, distance from health facility, women's autonomy in decision-making, perceived barriers to skilled birth services, and frequency of ANC attendance. Household wealth index, maternal age, and knowledge of danger signs during delivery were therefore not important determinants of SBA in the urban areas.

The parity of women was a significant determinant of utilization of SBA in both rural and urban areas. Rural women who had more than one birth were 11.9 times more likely to deliver with the assistance of SBAs than primiparous women (AOR = 11.88, CI: 3.76–37.56). Women of higher parity in urban areas were 4.0 times more likely (AOR = 4.19, CI: 1.91–9.20) to receive assistance from SBAs during delivery compared to primiparous women.

The likelihood of receiving skilled birth assistance during delivery is 34.9 and 3.9 times higher for women who attended ANC at least four times in rural and urban areas, respectively, compared to women who attended ANC less than four times (Table 3).

Distance from health facility had a significant influence on the utilization of skilled birth assistance in both rural and urban areas. Women who resided within 1–3 km of health facility were 6.0 and 9.6 times more likely to seek SBA in rural and urban areas, respectively, compared with their counterparts who live at least 4 km away from a health facility.

Women's autonomy was statistically associated with utilization of skilled delivery among women both in urban and rural areas. Women of higher decision-making autonomy were significantly more likely to have SBA than women of lower autonomy. Women of low perceived barriers to utilization of skilled delivery services in rural areas were 19.1 times more likely to patronize these services, compared with their counterparts who had high perceived barriers. Similar trend was reported in the urban areas, but then the probabilities were lower.

Strangely, women without knowledge of at least 3 danger signs during pregnancy were more likely to seek the services of skilled birth assistants in the rural areas, but this association was absent in the urban areas.

Maternal educational level and content of ANC were not strong determinants of SBA in both urban and rural women.

The set of predictors accounted for 67.9% and 46.3% of the variance in skilled birth attendance in rural and urban areas (Nagelkerke *R* square = 0.679 and 0.463), respectively.

### 3.5. Comparison of Differences in the Levels and Strength of Association of Selected Determinants of Skilled Birth Attendance in Rural and Urban Areas

Table 4 shows the determinants of skilled deliveries which differed in their levels across urban and rural areas. Strong differences in levels were observed with regard to maternal education, proximity to health facility, and frequency of ANC visits in favor of urban areas.

Women in urban areas are far more likely to have formal schooling than women in rural areas. The proportion of women with educational level of at least secondary school who sought SBA was significantly higher in the urban areas, compared to the rural areas.

The proportion of women who had SBA in urban areas and sought ANC services at least 4 times was greater than their counterparts in the rural areas (48.6% versus 37.5%).

There were also higher but insignificant proportions of women aged at least 35 years in urban areas who sought skilled birth services. The association between low perceived barriers (that is, perceived need for SBA) and skilled delivery services was significantly stronger in the rural than urban areas, but its level was lower in rural areas.

Table 4 also presents the results of the multivariate (logistic regression) analysis showing determinants of skilled delivery. The findings suggest that the determinants are not of the same strength in rural and urban areas. Separating the determinants of rural-urban disparity in SBA showed that the covariate effect is more dominant than the coefficient effect in the urban areas.

The key driving factors that contributed to the relatively high SBA coverage in the urban areas were higher frequency of ANC attendance of at least 4, distance from health facility less than 4 (km), and greater proportion of women attaining higher educational level of at least secondary school.

Women with higher educational level (secondary and higher) were significantly more in the urban areas (covariate effect) but the strength of association with SBA was not significant in both urban and rural areas.

In the case of deliveries assisted by SBAs, mothers aged at least 35 years were more likely to use the service compared with mothers of other age groups in only rural areas. This association was not significant in the urban areas. In rural areas, women with higher parity were more likely to use SBAs than primiparous women. This association was weaker in the urban areas.

Women of high autonomy were more likely to patronize skilled birth services in the rural areas, but this relationship was less among the urban women. A high level of women autonomy was rather common in the urban areas. The association between perceived barriers and uptake of skilled delivery services was stronger in the rural areas.

Distance from health facility less than 4 (km) was the greatest independent contributor to the variance in SBA in the urban areas. In the rural areas, frequency of ANC attendance was the greatest independent contributor to the variance in the disparity.

### 3.6. Binary Logistic Regression Analysis

Table 5 shows the determinants of skilled birth attendance (SBA) adjusted for confounding variables in both rural and urban areas combined. The odds of utilizing SBA are higher in urban areas compared with their rural counterparts. Women who live in urban areas have about 1.59 times higher odds of having SBA than those living in rural areas. Women of higher parity were significantly more likely to go for SBA than women of lower parity. Women with low perceived barriers (that is, higher perceived need) have 7.5 times higher odds of having SBA than those with lower perceived need. The other factors that are positively associated with having SBA are attending ANC four or more times and having high composite index of women's autonomy (CIWA) score.

## 4. Discussion

Though past studies have identified numerous determinants of skilled deliveries in rural and urban areas in many countries including Ghana [4, 25–27], very little is documented regarding how these determinants bring about rural-urban disparities in SBA. The uniqueness of the present study is the attempt to shed light on the effect of different levels of key determinants (that is, covariate effect) and their strength of association (that is, coefficient effect) on SBA outcomes.

Given the increasing rural-urban differences, understanding the relative importance of the various determinants of skilled delivery attendance in urban and rural areas in terms of whether they differ, is key to designing context-relevant programs and policy responses. An important health policy question that remains unanswered is whether different maternal health policies, interventions, and strategies are required in rural and urban areas. The answer to this question requires a good understanding of the main drivers of rural-urban disparities in SBA outcomes. Therefore, the main aim of this paper was to investigate which determinants of SBA are responsible for higher uptake of skilled birth services in urban areas of Northern Ghana.

To the best of our knowledge, the current study is the first to explain how exactly determinants contribute to rural-urban disparity in SBA in Northern Ghana, a finding that may be useful in other settings of similar characteristics. Differences in the level of the determinants (covariate effects) as well as differences in their strength of association with the outcome (coefficient effects) are key inputs for designing appropriate intervention measures.

Geographical differences in the determinants leading to rural-urban disparity in skilled delivery services may arise because of differences in the levels of determinants or differences in their strength of association with the expected outcomes (coefficient effects). Disparities may also arise from a combination of covariate and coefficient effects. If the differences arise largely due to covariate effects, similar policy frameworks and tools could be applied [12], but if differences are largely due to coefficient effects, then different strategies may be needed.

### 4.1. What Are the Determinants That Accounted for the Rural-Urban Differences in Skilled Delivery?

The results of the current study showed that though some determinants were common in both rural and urban settings, their levels and the strength of their associations with utilization of skilled delivery services across urban and rural areas varied substantially. For example, women of low perceived barriers to utilization of skilled delivery services in rural areas were 19.1 times more likely to patronize these services, compared with their counterparts who had high perceived barriers. Similar trend was reported in the urban areas, but then the probabilities were lower.

Strong differences in the levels of maternal education, proximity to health facility, and frequency of ANC visits were observed. The covariate effect was more dominant than the coefficient effect. This suggests that though these determinants were common in both areas, higher levels existed in favor of urban areas. For example, women in urban areas are far more likely to have formal schooling than women in rural areas.

There were also higher but insignificant proportions of women aged at least 35 years and women's decision-making power in urban areas who sought skilled birth services. The strength of association between low perceived barriers (that is, perceived need for SBA) and skilled delivery was significantly stronger in the rural than urban areas, but its level was lower in rural areas.

The key driving factors that contributed to the relatively high skilled delivery coverage in the urban areas were higher frequency of ANC attendance of at least 4, proximity (physical access) to health facility, and greater proportion of women attaining higher educational level of at least secondary school. Other factors of lesser extent were high perceived need for SBA and high decision-making power of women.

The role of maternal age as a determinant of SBA appears inconsistent in the literature; some literature suggests that women aged at least 35 years and having more than three children are less likely to use SBAs during pregnancies [28–30]. In our study, however, such women were more likely to utilize SBAs during delivery.

In earlier studies conducted in Ghana, women aged 35 years and above were more likely to deliver in a health facility, compared with their counterparts between 20 and 34 years in the rural areas [31, 32].

An overall composite index of women's autonomy (CIWA) comprising decision autonomy, movement autonomy, and maternal financial independence components was a significant predictor of uptake of skilled delivery assistance especially in the rural areas where coefficient effect was greater than in the urban areas. This finding concurs with recent studies in Ghana which indicated that women with health decision-making autonomy have higher tendency to health facility delivery as compared to those who are not autonomous [31, 33].

Autonomy is defined as the ability to obtain information and make decisions about one's own concerns [34]. Women's autonomy in healthcare decision-making is a recognized factor for better maternal and child health outcomes [35] and also serves as an indicator of women's empowerment. This assessment has been confirmed in some other studies which reported that when women are autonomous, their access to and utilization of healthcare improve [36–39]. Accordingly, it is being advocated that enhancing empowerment status of women in developing countries should be one of the strategies for improving maternal healthcare utilization [40].

In our sample, a greater proportion of women in urban areas who attended secondary or higher education received assistance from SBAs during delivery compared to those with no education. This finding concurs with a recent survey, the 2014 Ghana Demographic and Health Survey, which reported that women with secondary or higher education are more likely to seek skilled delivery services [4]. An earlier study that used the 2008 GDHS nationally representative sample reported that a woman's level of education is positively associated with institutional delivery only in the rural sample but not among urban women [31].

Furthermore, other studies conducted elsewhere including Turkey, Tanzania, and Bangladesh have stated that women's education is a strong determinant of the use of skilled assistance at delivery [36, 39–46].

The results underline the critical role education, especially that of women, plays in the promotion of maternal health. The fact supporting the role of education in this respect is that education serves as proxy for information and knowledge of available healthcare services [47]. Educational level is also a proxy for women's higher socioeconomic status that improves their ability to afford the cost of healthcare services [41]. Above all, education enhances level of autonomy of women and increases their decision-making power that results in improved freedom to make decisions including the use of maternal healthcare services [42, 48]. Furthermore, educated women are considered to have better knowledge and information on modern healthcare services [47, 49, 50].

Whereas some studies [4, 39, 44] have found a significant negative association between higher birth order and the use of SBAs at delivery in both urban and rural areas, this study did find a positive association between parity and uptake of skilled delivery services in only rural areas. The coefficient effect of parity was very strong in the rural areas.

A popular explanation in the literature for this association is that women with more children believe that they are more experienced to give birth safely and, hence, are less likely to use skilled assistance during delivery [44].

In this study household wealth index was associated with the utilization of skilled delivery services in the rural areas but not in the urban areas (Table 3), a finding that is consistent with one other study [43]. This can be explained by the fact that women living in the urban areas may not incur additional costs for transportation and other costs related to distance to access healthcare services. Distance from health facility less than 4 km was the greatest independent contributor to the variance in the disparity in the urban areas. A greater proportion of urban women were closer to health facilities than their rural counterparts. For the rural women, only those who can afford to pay transport related costs are able to visit health facilities.

Some other studies have reported that the likelihood of skilled delivery service utilization among women in households of high wealth index is higher compared to women of low wealth index among both urban and rural women [4, 39, 47, 51].

This study revealed that ANC utilization was a strong predictor of utilization of skilled assistance during delivery, a finding that is also consistent with findings of studies in other countries [43, 52–55]. As expected, the proportion of births delivered in a health facility increases substantially with increasing number of ANC visits.

Routine antenatal care services help raise awareness of safe delivery and give women familiarity with health services, and this can be strengthened through frequent visits.

The effect of perceived barriers on uptake of skilled birth services was significantly greater in the rural areas compared to the urban areas. Women of low perceived barriers to utilization of skilled delivery services in rural areas were 19.1 times more likely to patronize these services, compared with their counterparts who had high perceived barriers. Similar trend was reported in the urban areas, but then the probabilities were lower. Perceived barriers of women to skilled delivery services were therefore more effective in facilitating their perceived need for skilled delivery services in the rural areas than in urban areas.

Some other studies have found positive association between low perceived barriers (increased perceived need) and use of SBAs [32, 56, 57]. It is expected that reduced perceived barriers will lead to increased perceived need. Thus, the lower the perceived barrier index score, the greater the probability of perceived need for skilled birth assistance.

According to the Disparities in Skilled Birth Attendance (DiSBA) framework, the decision to use maternal health (MH) services, including use of SBAs, is based on three factors: perceived need for care, perceived accessibility (physical and financial) of the service, and perceived quality of the care [32]. These three factors are regarded as the proximal determinants of use of maternal health services.

Perceived need is influenced by women's current health status (e.g., having a pregnancy complication and the type and severity of the complication), reproductive factors (including age and parity), prior health status or pregnancy complications, and health knowledge (general and specific to pregnancy), as well as unknown factors that influence the development of pregnancy complications [58].

## 5. Conclusions

This study has identified the underlying factors accounting for rural-urban differences in skilled delivery in Northern Region of Ghana. The results of the current study showed strong differences in the levels of maternal education, proximity to health facility, and frequency of ANC visits. The covariate effect was more dominant than the coefficient effect. This suggests that, though these determinants were common in both areas, higher levels existed in favor of urban areas.

### 5.1. Recommendations and Policy Implications

The evidence suggests that urban-rural differences in SBA outcomes are primarily due to differences in the levels of critical determinants rather than the nature of the determinants themselves. Therefore, improving skilled delivery outcomes in this study population and other similar settings will not require different policy frameworks and interventions in dealing with rural-urban disparities in SBA outcomes. Context specific tailored approaches and strategies including targeting mechanisms, however, have to be designed differently.

Maternal health interventions aimed at removing rural-urban disparities in skilled birth services outcomes need to focus on the key determinants of SBAs identified in this study. In particular, there is an urgent need to bridge the maternal educational gaps between rural and urban areas, improve physical access to health facilities for deliveries, promote frequent ANC attendance during pregnancy, and embark on social behavior change to remove perceived barriers to SBA.

## Figures and Tables

**Table 1:** Comparison of baseline characteristics of the study groups.

Variable	Rural (*N* = 360)	Urban (*N* = 360)	Test statistic
	*n* (%)	*n* (%)	
Age (years)			
Under 25	122 (48.8)	128 (51.2)	Chi-squared (*χ*^2^) = 0.7, *p*=0.7
25–34	181 (51.6)	170 (48.4)	
At least 35	57 (47.9)	62 (52.1)	

Marital status			
Not married	32 (61.5)	20 (38.5)	*χ* ^*2*^ = 2.9, *p*=0.1
Married	328 (49.1)	340 (50.9)	

Educational level			
No formal education	294 (56.6)	225 (43.4)	*χ* ^*2*^ = 2.9, *p* < 0.001
Basic	46 (38.3)	74 (61.7)	
Secondary and above	20 (24.7)	61 (75.3)	

Parity			
1-2	176 (47.6)	194 (52.4)	*χ* ^*2*^ = 1.8, *p*=0.4
3-4	117 (52.5)	106 (47.5)	
>4	67 (52.8)	60 (47.2)	

Distance from health facility (km)			
1–3	92 (55.1)	75 (44.9)	*χ* ^*2*^ = 2.3, *p*=0.1
At least 4 km	268 (48.5)	285 (51.5)	

Household wealth index			
Low	195 (48.5)	207 (51.5)	*χ* ^*2*^ = 0.8, *p*=0.4
High	165 (51.9)	153 (48.1)	

Religion			
Islam	202 (49.6)	205 (50.4)	*χ* ^*2*^ = 68.1, *p* < 0.001
Christianity	89 (37.4)	149 (62.6)	
African traditional religion (ATR)	69 (92.0)	6 (8.0)	

**Table 2:** Levels of utilization of skilled delivery services among women in rural and urban areas.

Predictor variable	Utilization of SBA in rural areas *n* (%)		Test statistic	Utilization of SBA in urban areas *n* (%)		Test statistic
Age (years)	No	Yes		No	Yes	
Under 25	95 (77.9)	27 (22.1)	Chi-squared (*χ*^2^) = 35.9, *p* < 0.001	81 (63.3)	47 (36.7)	*χ* ^2^ = 14.2, *p*=0.001
25–34	96 (53.0)	85 (47.0)		82 (48.2)	88 (51.8)	
At least 35	19 (33.3)	38 (66.7)		22 (35.5)	40 (64.5)	

Educational level						
No formal education	184 (62.6)	110 (37.4)	*χ* ^2^ = 14.4, *p*=0.001	119 (52.9)	106 (47.1)	*χ* ^2^ = 0.6, *p*=0.7
Basic	21 (45.7)	25 (54.3)		37 (50.0)	37 (50.0)	
Secondary and above	5 (25.0)	15 (75.0)		29 (47.5)	32 (52.5)	

Parity						
1-2	127 (72.2)	49 (27.8)	*χ* ^2^ = 29.1, *p* < 0.001	116 (59.8)	78 (40.2)	*χ* ^2^ = 12.8, *p*=0.002
3-4	58 (49.6)	59 (50.4)		47 (44.3)	59 (55.7)	
>4	25 (37.3)	42 (62.7)		22 (36.7)	38 (63.3)	

Distance from health facility (km)						
1–3	23 (25.0)	69 (75.0)	*χ* ^2^ = 56.5, *p* < 0.001	9 (12.0)	66 (88.0)	*χ* ^2^ = 58.8, *p* < 0.001
At least 4 km	187 (69.8)	81 (30.2)		176 (61.8)	109 (38.2)	

Household wealth index						
Low	125 (64.1)	70 (35.9)	*χ* ^2^ = 5.8, *p*=0.02	113 (54.6)	94 (45.4)	*χ* ^2^ = 1.9, *p*=0.2
High	85 (51.5)	80 (48.5)		72 (47.1)	81 (52.9)	

ANC content						
Inadequate (<median score of 5)	120 (64.9)	65 (35.1)	*χ* ^2^ = 6.7, *p*=0.01	92 (55.8)	73 (44.2)	*χ* ^2^ = 2.3, *p*=0.1
Adequate (median score of at least 5)	90 (51.4)	85 (48.6)		93 (47.7)	102 (52.3)	

Classification of women autonomy						
Low	130 (66.7)	65 (33.3)	*χ* ^2^ = 12.2, *p* < 0.001	110 (57.3)	82 (42.7)	*χ* ^2^ = 5.7, *p*=0.02
High	80 (48.5)	85 (51.5)		75 (44.6)	93 (55.4)	

Classification of perceived barriers						
Low inadequate (<median score of 2)	96 (42.7)	129 (57.3)	*χ* ^2^ = 60.6, *p* < 0.001	81 (36.3)	142 (63.7)	*χ* ^2^ = 53.2, *p* < 0.001
High (median score of at least 2)	114 (84.4)	21 (15.6)		104 (75.9)	33 (24.1)	

ANC visits during previous pregnancy						
Less than 4	174 (77.3)	51 (22.7)	*χ* ^2^ = 89.1, *p* < 0.001	124 (67.0)	61 (33.0)	*χ* ^2^ = 37.3, *p* < 0.001
At least 4	36 (26.7)	99 (73.3)		61 (34.9)	114 (65.1)	

Knowledge of danger signs during pregnancy						
No	42 (58.3)	30 (41.7)	*χ* ^2^ = 0.0, *p*=1.0	41 (64.1)	23 (35.9)	*χ* ^2^ = 5.0, *p*=0.03
Yes	168 (58.3)	120 (41.7)		144 (48.6)	152 (51.4)	

^*∗*^Significant at *p* < 0.05; ^*∗∗*^significant at *p* < 0.01; ^*∗∗∗*^significant at *p* < 0.001.

**Table 3:** Strength of association (coefficient effect) of SBA determinants across urban and rural areas.

Predictor	(Rural Model)		(Urban Model)	
	Odds ratio	95% confidence interval (CI)	Odds ratio	95% confidence interval (CI)
Age (years)				
Under 25	Reference	Reference	Reference	Reference
25–34	1.21	CI: 0.52–2.85	0.76	CI: 0.39–1.47
At least 35	9.80	CI: 2.23–43.06^*∗∗*^	1.74	CI: 0.67–4.49

Educational level				
No formal education	Reference	Reference	Reference	Reference
Basic	0.79	CI: 0.28–2.24	1.00	CI: 0.51–1.96
Secondary and above	2.40	CI: 0.55–10.39	1.22	CI: 0.60–2.50

Parity				
1	Reference	Reference	Reference	Reference
>1	11.88	CI: 3.76–37.56^*∗∗∗*^	4.20	CI: 1.92–9.21^*∗∗∗*^

Distance from health facility (km)				
1–3	6.05	CI: 2.54–14.39^*∗∗∗*^	9.62	CI: 3.78–24.49^*∗∗∗*^
At least 4 km	Reference	Reference	Reference	Reference

Household wealth index				
Low	Reference	Reference	Reference	Reference
High	2.37	CI: 1.09–5.89^*∗*^	0.89	CI: 0.45–1.76

ANC content				
Inadequate	Reference	Reference	Reference	Reference
Adequate	0.67	CI: 0.30–1.47	0.77	CI: 0.44–1.38

Classification of women autonomy				
Low	Reference	Reference	Reference	Reference
High	8.24	CI 3.38–20.08^*∗∗∗*^	2.65	CI: 1.41–4.97^*∗∗*^

Classification of perceived barriers				
Low	19.08	CI: 7.27–50.08^*∗∗∗*^	4.22	CI: 2.38–7.49^*∗∗∗*^
High	Reference	Reference	Reference	Reference

ANC visits during previous pregnancy				
Less than 4	Reference	Reference	Reference	Reference
At least 4	34.86	CI: 12.74–95.38^*∗∗∗*^	3.92	CI: 2.24–6.86^*∗∗∗*^

Knowledge of danger signs during pregnancy				
No	2.91	CI: 1.03–8.19^*∗*^	0.78	CI: 0.37–1.64
Yes	Reference	Reference	Reference	Reference

(Pseudo) *R*^2^	70.8%		43.7%	

^*∗*^Significant at *p* < 0.05; ^*∗∗*^significant at *p* < 0.01; ^*∗∗∗*^significant at *p* < 0.001.

**Table 4:** Comparison of differences in the level and strength of association of selected determinants of skilled delivery attendance stratified by residence type.

Predictor variable	Level of predictor in districts (%)		Difference in predictor level (%)	Strength of association between determinant and SBA (AOR (CI))		Is the effect covariate or coefficient in urban areas?
	Rural	Urban	Urban-rural	Rural	Urban	
% of women aged at least 35 years	15.8	17.2	4.2	9.80 (2.23, 43.06)^*∗∗*^	1.74 (0.67, 4.49)	None
Educational level of at least secondary school	5.6	16.9	11.3^*∗∗∗*^	2.40 (0.55, 10.39)	1.22 (0.60, 2.50)	Covariate
Parity > 1	72.5	69.7	−2.8	11.88 (3.76, 37.56)^*∗∗∗*^	4.20 (1.92, 9.21)	None
Distance from health facility less than 4 (km)	20.8	25.6	4.8^*∗*^	6.05 (2.54, 14.39)^*∗∗∗*^	9.62 (3.78, 24.49)^*∗∗∗*^	Both covariate and coefficient
High household wealth index	45.8	42.5	−3.3	2.37 (1.09, 5.89)^*∗*^	0.89 (0.45, 1.76)	None
Adequate ANC content	48.6	54.2	5.6	0.67 (0.30, 1.47)	0.77 (0.44, 1.38)	None
High women autonomy	45.8	49.8	3.0	8.24 (3.38, 20.08)^*∗∗∗*^	2.65 (1.41, 4.97)	None
Low perceived barriers	37.5	41.1	3.6	19.08 (7.27, 50.08)^*∗∗∗*^	4.22 (2.38, 7.49)^*∗∗∗*^	None
ANC visits of at least 4	37.5	48.6	11.1^*∗∗*^	34.86 (12.74, 95.38)^*∗∗∗*^	3.92 (2.24, 6.86)^*∗∗∗*^	Covariate

^*∗*^Significant at *p* < 0.05; ^*∗∗*^significant at *p* < 0.01; ^*∗∗∗*^significant at *p* < 0.001. AOR (CI): adjusted odds ratio with 95% confidence interval.

**Table 5:** Determinants of skilled delivery (logistic regression).

	B	SE	Wald	Sig.	Exp (*β*)	95% CI for Exp (*β*)	
						Lower	Upper
Frequency of ANC visits, at least 4	2.039	0.216	89.47	<0.001	7.69	5.04	11.73
Parity (reference: 1-2)			47.11	<0.001			
3-4	1.066	0.238	20.12	<0.001	2.91	1.82	4.63
>4	2.177	0.335	42.21	<0.001	8.82	4.57	17.01
Distance from health facility <4 km	1.670	0.260	41.24	<0.001	5.31	3.19	8.85
High women autonomy	0.947	0.215	19.42	<0.001	2.58	1.69	3.93
Low perceived barriers	2.016	0.232	75.76	<0.001	7.51	4.77	11.83
Urban residence	0.462	0.203	5.15	0.023	1.59	1.07	2.37
Constant	−4.208	0.358	138.15	<0.001	.015		

The set of predictors accounted for 53.6% of the variance in skilled birth attendance (Nagelkerke *R* square = 0.536).

## Data Availability

The quantitative data presented in the form of SPSS used to support the findings of this study are included within the supplementary information file(s).

## Additional Points

*Limitations of This Study*. The study design has some limitations that should be considered when interpreting the results. Since this was a cross-sectional survey study, it was not possible to draw causal conclusions about the determinants of skilled delivery services utilization. However, many of the associations we found are well established in the literature [16, 59–61] and are biologically plausible. Furthermore, the cross-sectional design limits the ability to determine how the district inequalities evolve over time.

## Supplementary Materials

Supplementary Materials The Supplementary Materials include the values of the dataset on rural-urban utilization.
